# Impact of HIF-1α, LOX and ITGA5 Synergistic Interaction in the Tumor Microenvironment on Colorectal Cancer Prognosis

**DOI:** 10.3390/diagnostics15020184

**Published:** 2025-01-14

**Authors:** Hayriye Tatlı Doğan, Mehmet Doğan, Seda Kahraman, Doğukan Çanakçı, Mehmet Ali Nahit Şendur, Mustafa Tahtacı, Fazlı Erdoğan

**Affiliations:** 1 Department of Pathology, Faculty of Medicine, Ankara Bilkent City Hospital, Ankara Yıldırım Beyazıt University, Ankara 06800, Turkey; 2 Department of Medical Oncology, Ankara Yıldırım Beyazıt University, Ankara 06800, Turkey; 3 Faculty of Medicine, Ankara Yıldırım Beyazıt University, Ankara 06800, Turkey; 4 Department of Gastroenterology, Faculty of Medicine, Ankara Bilkent City Hospital, Ankara Yıldırım Beyazıt University, Ankara 06800, Turkey

**Keywords:** hypoxia, colorectal cancer, microenvironment

## Abstract

**Background**: As colorectal cancers are histopathologically and molecularly highly heterogeneous tumors, it is necessary to consider the tumor’s microenvironment as well as its cellular characteristics in order to determine the biological behavior of the tumor. This study included 100 patients who underwent resection for colorectal cancer. We aimed to investigate the relationships between the expression status of the HIF-1α, LOX and ITGA5 proteins and clinicopathologic parameters. **Methods**: HIF-1α, LOX and ITGA5 antibodies were applied immunohistochemically to tissue microarrays prepared from tumor samples. Expression status in the tumor microenvironment were evaluated using a combined scoring system based on staining intensity and the percentage of positively stained cells. Nuclear HIF-1α expression in tumor cells was quantified, with >1% considered positive. The staining of HIF-1α, ITGA5 and LOX was analyzed in relation to prognostic and molecular features. **Results**: The staining of HIF-1α, ITGA5 and LOX in the tumor microenvironment demonstrated a positive correlation with one another and with HIF-1α and LOX expression in tumor cells. In patients with KRAS, NRAS or BRAF mutation and the moderate to strong expression of all three of these proteins in the tumor microenvironment, the number of metastatic lymph nodes was higher than in other patients. Stage IV patients with the moderate to strong expression of HIF-1α, ITGA5 or LOX in the microenvironment had lower progression-free survival than those with weak expression (*p* < 0.05). In addition, female gender; moderate to strong HIF-1α, LOX and ITGA5 stromal expression; and metastatic first line chemotherapy only were found to be independently associated with an increased risk of progression. **Conclusions**: These markers may be useful in predicting treatment responses and may also guide the development of alternative or combined treatments that specifically target molecules such as HIF and LOX. Our study should be supported by more comprehensive studies addressing the tumor stroma and its prognostic importance.

## 1. Introduction

Colorectal cancers (CRCs) account for a significant proportion of cancer-related mortality and morbidity [1] with five-year overall survival in metastatic CRC is around 15%. Metastasis occurs in 33% of patients at baseline or during follow-up [2]. CRC is a heterogeneous disease resulting from a combination of well-characterized genetic and epigenetic alterations. Therefore, treatments for this heterogeneous group of diseases have begun to vary based on the biological characteristics of the tumor and its stage. In addition to conventional chemotherapy, molecularly targeted drugs have enabled the development of treatment options for CRCs [3]. Molecular prognostic markers are important in predicting patients’ responses to treatment or determining different treatment options [4,5]. Intracellular regulation disorders such as those affecting the growth factor signaling pathway, apoptosis and DNA repair mechanisms have been associated with chemoresistance in tumors [6]. KRAS activation mutations are found with a frequency of 35–45% in CRC, which serve as a predictive marker for resistance to anti-epidermal growth factor receptor (EGFR) therapy [7,8].

In addition to tumor cells, changes in the tumor microenvironment (TME), also play important roles in cancer progression, metastasis and treatment resistance. The TME consists of immune cells, stromal cells and extracellular matrix. The dynamic and reciprocal interaction between cancer cells and the TME support cancer cell survival, local invasion and metastasis [9]. Extracellular matrix (ECM) remodeling plays an important role in tumor development, invasion and metastasis [10]. Matrix metalloproteinases are responsible for matrix degradation, while lysyl oxidase (LOX) is responsible for matrix stiffness. These molecules also exert broad effects on immune cells, mesenchymal cells, epithelial cells, cancer cells and stem cells [11]. Indeed, there is growing evidence that the effect of the ECM on treatment resistance is increasingly important. It has been suggested that the ECM creates a protective barrier for anti-cancer drugs or blocks apoptosis through the activation of growth signaling pathways or integrin-like transmembrane receptors [12,13]. Integrins are mainly expressed on the cell membrane surface and are involved in cell–cell and cell–matrix interactions. Integrins are also present in the cytoplasm and facilitate interactions between the ECM and intracellular structures. The effects of integrins on tumors are mediated by cytokines, ligands and angiogenesis. ITGA5 is one of the members of the integrin family which is a fibronectin receptor that is important in ECM and intracellular signaling interactions [14]. ITGA5 is closely associated with the focal adhesion kinase (FAK), AKT and PI3K signaling activation. These properties strengthen the potential of ITGA5 as a biomarker and therapeutic target. ITGA5 has been shown to be associated with proliferation, invasion and metastasis in many cancers [15,16], and it has been shown that the activation of integrin signaling is related to resistance to therapy and the acquisition of metastatic traits.

Hypoxia-inducible factor-1 alpha (HIF-1α) has an emerging role in the chemo/radioresistance of tumors and the complex mechanisms through which HIF-1 mediates resistance to anti-cancer therapies are gradually being elucidated. HIF-1 activates the transcription of genes involved in key aspects of cancer biology, and regulates the expression of many downstream genes, including those involved in hypoxic energy metabolism, angiogenesis, matrix remodeling, autophagy and cell invasion/migration [17,18]. HIF-1α, encoded by the HIF-1α gene, activates the transcription of enzymes involved in ECM remodeling, particularly collagen prolyl and lysyl hydroxylase (LOX). Considering the roles of integrins and LOX in matrix stiffness, it has been reported that resistance may develop due to the difficulty of chemotherapy agents reaching the tumor. Recent studies have emphasized that the activation of the HIF-1α signaling pathway is especially increased in chemotherapy-resistant breast cancers, indicating that it may be associated with resistance. Due to the role of hypoxia in ECM modeling, it has been shown through cell culture experiments that HIF-1α can activate integrin and focal adhesion. It has been reported that HIF-1α and LOX expression affects in invasion and metastasis and is related to a more aggressive clinical course [6].

Intestinal epithelium-derived HIF is associated with Wnt/β-catenin activity, inflammation and tumor-specific metabolism, whereas myeloid HIF is essential for tumor-associated fibroblast activation [19]. Taken together, these findings provide compelling evidence that LOX and HIF-1α act in synergy to promote tumorigenesis and suggest that the reciprocal regulation of HIF-1α and LOX is a crucial mechanism for the adaptation of tumor cells to hypoxia [20]—hypoxia induces HIF-1α and HIF-1α activates LOX transcription. Then, on one hand, LOX increases the expression of ITGA5 and its ligand fibronectin, and on the other hand, it also induces collagen cross-linking. This may reduce the penetration of chemotherapeutic drugs into the cell. The hypoxia-induced downregulation of some microRNAs (e.g., miR-142-3p) that regulate the HIF-1α, LOX and ITGA5 axis has been shown to cause HIF-1α, LOX, ITGA5 and related FAK/Src signaling activation. This may lead to blockade of drug-induced apoptosis and chemoresistance. For this reason, it has been reported that the inhibition of LOX, FAK and SRC may overcome CT resistance [21].

In addition, studies have suggested that a positive feedback loop between oncogenic KRAS and HIF-1α contributes to drug resistance in CRC [22]. The limited number of preclinical studies have reported that RAS inhibitors, which have recently come to the fore, especially in cases with KRAS mutation, inhibit LOX and HIF-1α, thus preventing progression and metastasis [23].

The aim of this study is to investigate the interactions of the molecules HIF-1α, LOX and ITGA 5 key players in tumor microenvironment and ECM remodeling—whose roles and interplay in drug resistance have been previously reported in preclinical studies. Specifically, this study seeks to examine these interactions in human colorectal cancer tissues and explore their relationship with prognostic parameters, particularly progression-free survival, as well as their association with molecular characteristics.

## 2. Materials and Methods

### 2.1. Selection of Cases and Tissue Microarray Creation

This study included 100 patients older than 18 years of age who underwent surgery for colorectal carcinoma and for whom somatic mutation analysis for KRAS, NRAS and BRAF could be performed between February 2019 and January 2021. Inclusion criteria required patients to have a histologically confirmed diagnosis of adenocarcinoma, availability of formalin-fixed paraffin-embedded tissue samples and clinical–pathological data. Exclusion criteria included incomplete medical records or inadequate tissue samples for mutation analysis.

This study was approved by the Ethics Committee of Ankara City Hospital, dated 11 January 2023 and numbered E1-23-3181.

Parameters such as age, gender, tumor location, size, histological grade, lymph node metastasis, lymphovascular invasion (LVI), perineural invasion (PNI), stage at diagnosis, progression date, death date and mismatch repair (MMR) protein expression status were obtained from pathology reports and hospital databases.

Overall survival was calculated as the time from the date of initial diagnosis to the date of death or last follow-up. In stage IV patients, progression-free survival (PFS) was defined as the time to first progression or deaths related to progression [24,25].

From hematoxylin and eosin (H&E)-stained preparations representing the tumor, an appropriate tumor area was selected to prepare tissue microarray (TMA) blocks. Two samples with a diameter of 3 mm, which best represent the tumor and the infiltrative border of the tumor, were removed from the block with the Quick-Ray^®^ (Unitma Co., Seongnam-si, Republic of Korea) Manual Tissue Microarrayer device, and TMA blocks were created.

### 2.2. Immunohistochemistry (IHC) Procedure

Antibodies for LOX (EPR4025, Rabbit Monoclonal IgG, 1:300), (EPR7854, ITGA5 (Rabbit Monoclonal IgG, 1:100) and HIF-1α (EP1215Y, Rabbit Monoclonal IgG, 1:300) were used for immunohistochemical analysis. Each antibody (either mouse or rabbit) was optimized with known positive controls according to datasheets.

Next, 4 μm thick sections were prepared from the TMA blocks and immunohistochemical staining was performed in a Roche Ventana Ultra device. The deparaffinization step is performed in the Ventana platform using EZ prep solution. For each antibody, optimal pre-treatment consisting of antigen retrieval was performed with CC1 (EDTA buffer, pH 8) at 95 °C for 36 min. Then, sections were incubated with primary antibodies (HIF-1α, ITGA5, LOX) at 37 °C for 30 min. Antigen–antibody reactions were observed using the Ventana UltraView Universal DAB Detection (Tucson, Arizona, USA). Counterstaining was performed using Ventana Hematoxylin II for 8 min, followed by the bluing of the reagent for 4 min. Slides were rinsed with distilled water, passed through graded alcohols and xylene, dried, sealed with balsam and prepared for microscopic analysis. Formun ÜstüFormun Altı.

### 2.3. IHC Score

**Figure 1:**
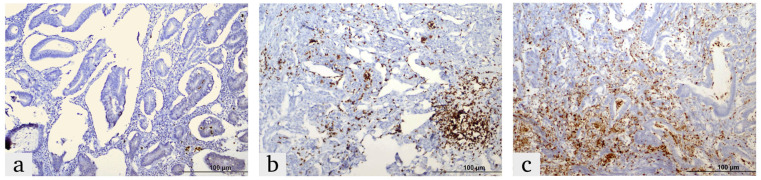
Representative images of HIF-1α expression in colorectal cancer tissues, demonstrating the expression in the tumor microenvironment and tumor cells: (**a**) weak, (**b**) moderate and (**c**) strong.

**Figure 2:**
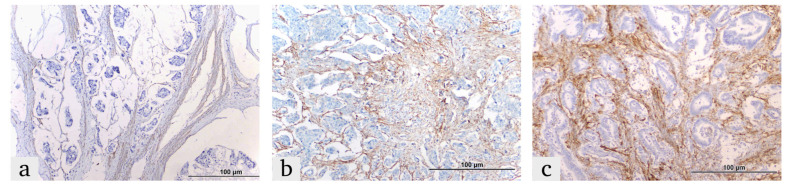
Representative images of ITGA5 expression in colorectal cancer tissues, demonstrating the expression in the tumor microenvironment: (**a**) weak, (**b**) moderate and (**c**) strong.

**Figure 3:**
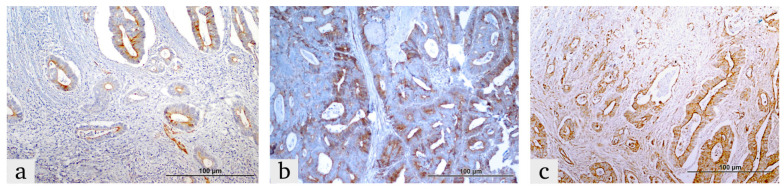
Representative images illustrating LOX expression in colorectal cancer tissues within the tumor microenvironment and tumor cells. LOX expression in the tumor microenvironment is categorized as (**a**) weak, (**b**) moderate and (**c**) strong. In tumor cells, LOX expression is shown as (**a**) low and (**b**,**c**) high expression levels.

The expression of HIF-1α, ITGA5 and LOX in the tumor microenvironment was evaluated based on a combination of staining intensity and the percentage of positively stained cells [26,27]. (Figure 1, Figure 2 and Figure 3).

Expression was classified as:

Weak Positive (+1): Weak reaction in less than 10% of the tumor microenvironment.

Moderate Positive (+2): Weak-to-moderate reaction in 10–50% of the tumor microenvironment.

Strong Positive (+3): Strong reaction in more than 50% of the tumor microenvironment

The nuclear expression of HIF-1α in epithelial tumor cells was quantified as a percentage of positive cells, and cases with expression above 1% were considered positive [28].

LOX expression was also evaluated in tumor cells. Cytoplasmic LOX expression in the epithelial component of the tumor was scored as:

1+ (Mild), 2+ (Moderate) or 3+ (Strong) based on intensity.

The percentages of positively stained cells were scored as:

- 0 (0%), 1 (0–25%), 2 (25–50%), 3 (50–75%) and 4 (75–100%).

The final LOX score was calculated by multiplying the percentage score by the intensity score. Cases with a final score of ≤4 were classified as having low LOX expression, while those with a score >4 were classified as having high LOX expression [29] (Figure 3).

### 2.4. Single Gene Real-Time PCR Tests for KRAS, NRAS and BRAF

EasyPGX KRAS, NRAS and BRAF mutation detection kits were used in the real-time PCR platform. After manually macro-dissecting the tumor areas and isolating DNA, we detected target mutations including KRAS, NRAS codon 12, 13, 59, 61, 117 and 146 and BRAF kodon 600: V600E (1799T>A), V600Ecomplex (1799_1800TG>AA), V600K (1798_1799GT>AA), V600D (1799_1800TG>AT) and V600R (1798_1799GT>AG) using real-time PCR reactions. The results were analyzed using the EasyPGX^®^ software (version 4.0.13, Brescia, Italy).

## 3. Statistical Analysis

All analyses were performed in IBM SPSS v25.0 (IBM Corp., Armonk, NY, USA). The conformity of quantitative variables to a normal distribution was evaluated via the Shapiro–Wilk test. Descriptive statistics are given as mean ± standard deviation for normally distributed variables, median (minimum–maximum value) for non-normally distributed variables and frequency (percentage) for qualitative variables. Survival times were calculated using the Kaplan–Meier method. A log rank test was used to compare survival times between groups. Prospective multiple Cox regression analysis was used to determine the factors independently associated with mortality and progression. The Spearman correlation coefficient was used to analyze the correlations between protein expressions. A Student’s t test or one-way analysis of variance (ANOVA) was used to analyze quantitative variables that were suitable for normal distribution according to the number of groups. Meanwhile, Mann–Whitney U test or Kruskal–Wallis test was used to analyze quantitative variables that were not normally distributed according to the number of groups. The chi-square test, Fisher’s exact test or Fisher–Freeman–Halton tests were used to analyze qualitative variables. Bonferroni correction was applied for pairwise comparisons. A value of *p* < 0.05 values was considered statistically significant.

## 4. Results

### 4.1. Patient and Tumor Characteristics

The study included 100 patients (35 women and 65 men). The mean age at diagnosis was 61.77 ± 11.43 (range 32–85) years. The most common location was the left colon (52.0%). A total of 49 (49.0%) patients had distant organ metastasis at diagnosis and classified as stage IV. Among the 49 patients with distant organ metastases, a total of 53 metastatic sites were identified, as some patients had metastases in more than one organ. A total of thirty-eight patients had liver metastases, three patients had both liver and ovarian metastases, one patient had both liver and peritoneal metastases, two patients had ovarian metastases, three patients had peritoneal metastases, one patient had lung metastases and one patient had bladder metastases. In terms of T staging, 33 patients were T4, 6 were T2 and 61 were T3. Sixty-nine (69.0%) patients had LVI and forty (40.0%) had PNI. The median follow-up period was 36 (range 3 to 66) months. In addition, 13 of the 100 cases were MMR deficient. KRAS mutation was detected in 30% and BRAF mutation was detected in 11% of the patients (Table 1).

### 4.2. Overall Survival Analysis and Correlation with Clinicopathological Findings

The mean survival time for all patients was calculated as 40.71 (95% CI: 35.82–45.60) months. The survival time was lower in female patients than in male patients (*p* = 0.006). There were no significant differences in overall survival between patients receiving metastatic first-line CT (folinic acid, oxoliplatin, irinotecan combination), CT + Anti-EGFR and CT + Anti VEGF (*p* = 0.313). No significant correlation was found between HIF-1α, LOX or ITGA 5 expression in TME or tumor cells and overall survival. 

### 4.3. Progression-Free Survival Analysis and Correlation with Clinicopathological Findings

In the follow-up of 49 patients with stage 4 from baseline, 28 of the 40 patients who could be evaluated progressed, while no progression was observed in the remaining 12 patients. In these patients, the mean progression-free survival time was 11.07 (95% CI: 8.4–14.09) months and the median was 8.23 months (95% CI 6.02–10.43). PFS was lower in women than men (*p* = 0.016).

PFS was significantly lower in BRAF mutation-positive patients compared to BRAF wild-type patients (*p* = 0.001).

PFS was significantly lower in HIF-1α tumor-positive cases compared to HIF-1α tumor negative ones (*p* = 0.030).

**Figure 4:**
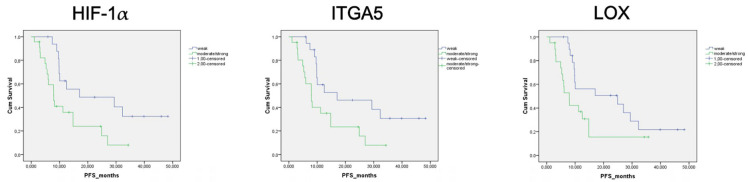
Progression-free survival curve according to HIF-1α, ITGA5 and LOX expression status in tumor microenvironment.

Regarding the expression of HIF-1α, ITGA5 and LOX in the TME, the median PFS was significantly higher in cases with weak expression compared to those with moderate or strong expression. The median PFS was 17.1 months for cases with weak HIF-1α expression, 7.93 months for those with moderate expression and 7.97 months for those with strong expression. For ITGA5 expression, the median PFS was 17.1 months for weak expression, 8.2 months for moderate expression and 5.6 months for strong expression. The median PFS was 24.9 months for cases with weak LOX expression, 7.9 months for moderate expression and 6.1 months for strong expression. Due to the limited sample size and the similar median PFS durations observed among patients with moderate and strong expression, survival analysis was conducted by grouping weak expression as one group and moderate-to-strong expression as another group. The PFS was significantly lower in cases with a moderate to strong HIF-1 expression in the tumor microenvironment compared to those with weak HIF-1α expression in the TME (*p* = 0.013). Patients with moderate to strong ITGA5 expression in the TME had significantly lower PFS compared to those with weak expression (*p* = 0.018). PFS was also lower in patients with moderate to strong LOX expression than in patients with weak LOX expression in the TME (*p* = 0.05) (Figure 4). However, there was no significant difference in PFS between patients with high and low LOX expression in tumor cells (*p* = 0.572).

**Figure 5:**
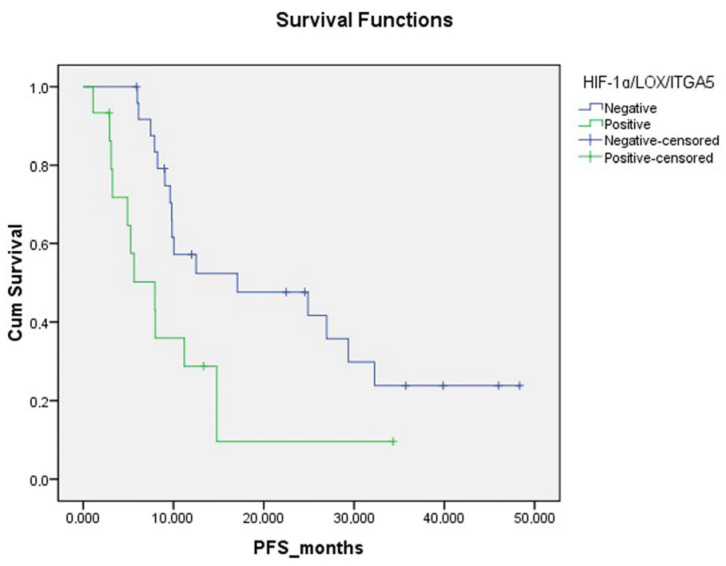
Progression-free survival curve according to triple HIF-1α/LOX/ITGA5 expression status in tumor microenvironment.

**Figure 6:**
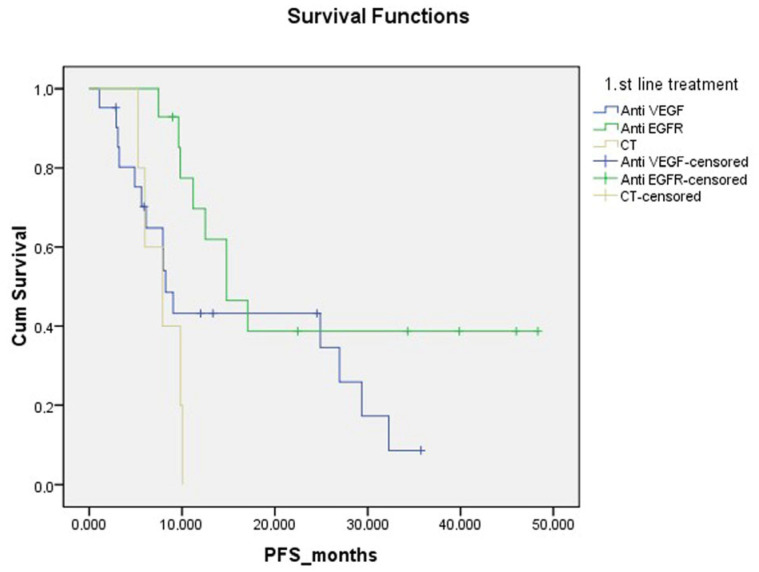
Progression-free survival curve according to first-line treatment.

Progression-free survival was significantly lower in patients with moderate to strong expression of all three HIF-1α, LOX and ITGA5 IHCs in the TME. (*p* = 0.014, Figure 5). PFS was significantly lower in BRAF mutation-positive patients than in other patients (*p* = 0.001). PFS was significantly lower in patients who received only CT as a metastatic first-line treatment compared to patients who received anti-EGFR and anti-VEGF (*p* = 0.023) (Figure 6, Table 2).

The study conducted a Cox regression analysis using the variables that were found to be statistically significant in the log rank test. These variables included female gender (HR: 0.350; 95% CI: 0.145–0.846; *p* = 0.020), moderate to strong HIF-1/LOX/ITGA5 (HR: 4.324; 95% CI: 1.291–14.481; *p* = 0.018), metastatic first-line CT only (*p* = 0.018) and metastatic first-line treatment (*p* = 0.023). First-line CT alone (*p* = 0.048) and CT + Anti-VEGF were independently associated with an increased risk of progression (*p* = 0.048) (Table 3).

**Figure 7:**
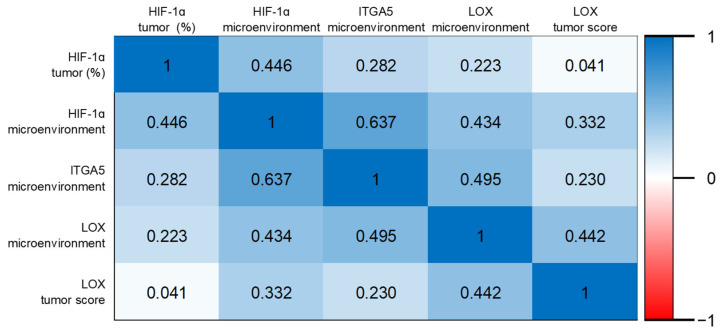
Heat map of Spearman’s correlation coefficients.

Positive correlations were found between HIF-1α tumor positivity and ITGA5 microenvironment (r = 0.282; *p* = 0.004); HIF-1α microenvironment and ITGA5 microenvironment (r = 0.637; *p* < 0.001); HIF-1α microenvironment and LOX microenvironment (r = 0.434; *p* < 0.001); HIF-1α microenvironment and LOX tumor score (r = 0.300; *p* = 0.002); HIF-1α microenvironment and LOX tumor score (r = 0.332; *p* = 0.001); ITGA5 microenvironment and LOX tumor score (r = 0.245; *p* = 0.014); and ITGA5 microenvironment and LOX tumor outcome (r = 0.230; *p* = 0.022) (Figure 7).

The number of metastatic lymph nodes was higher in mutation-positive (KRAS, NRAS or BRAF) and moderately to strongly triple IHC (HIF-1α, LOX and ITGA5) (+2 and +3)-positive patients compared to other patients (*p* = 0.033). Well differentiation status was lower in mutation and all IHC-positive patients (moderate to strong) than in other patients (*p* = 0.026). The percentage of stage N0 was also lower in these patients than in other patients (*p* = 0.028). In patients who had mutations and were all IHC (+2 and +3)-positive, the absence of LVI was found to be lower than in other patients (*p* = 0.021, Table 4).

## 5. Discussion

In this study, we explored the prognostic impacts of HIF-1α, LOX and ITGA5 expression in the tumor microenvironment and their relationship with KRAS/NRAS/BRAF mutation status in colorectal cancers. HIF molecules are critical mediators of tissue hypoxia which have recently become a target in cancer therapy. HIF and hypoxia signaling activate many signaling pathways, such as cyclins, MTOR and VEGF [30,31], and cellular HIF levels may modify the response to therapies targeting these pathways. Large cohort studies have shown that HIF-1α expression is associated with a high mortality rate independent of molecular variables and patient characteristics [32]. While HIF-1α and HIF-2α expressions are generally associated with poor prognosis in colorectal cancers, some studies have failed to establish their independent prognostic value. Hypoxia determinants such as HIF-1α and HIF-2α are found in both the tumor epithelium and tumor-associated stroma. There are few studies in the literature considering stromal HIF expression [19,33,34]. HIF-1α is degraded as a result of upregulation of prolyl hydroxylase domain proteins after prolonged hypoxia. Moreover, a decrease in HIF expression may also occur due to nutrient depletion [35]. Therefore, epithelial HIF expression may not fully reflect tumor hypoxia. Stromal HIF-2α expression, in contrast, has been associated with reduced overall survival and identified as an independent prognostic factor [33]. Our study found that increased HIF-1α expression in the tumor microenvironment was correlated with decreased progression-free survival (PFS), although stromal HIF expression showed no significant impact on overall survival.

There is an important interaction between oncogenic KRAS and hypoxia; mutant KRAS enhances HIF-1α translation via the PI3K pathway, while mutant BRAF regulates the mRNA expression and translation of HIF isoforms. This suggests that such differential regulation contributes to the different phenotypic characteristics of KRAS and BRAF mutant tumors. It has also been found that the rate of HIF-1α protein synthesis was high in a KRAS mutant cell culture [36].

LOX, an extracellular matrix regulator, plays a pivotal role in cancer metastasis. Baker et al. have shown that LOX increases cell proliferation, matrix stiffness and invasion in colorectal cancers in vitro and is associated with metastasis in vivo [37]. LOX-associated ECM stiffness may also be linked to integrin receptor activation [38]. Treatment strategies combining LOX inhibition with CT have shown promise in disrupting microenvironment-related survival signals. LOX inhibition has been shown to reduce ECM stiffness, suppress ITGA5 expression, induce apoptosis and combat chemoresistance [21]. Additionally, LOX inhibition facilitates T-cell migration and enhances the efficacy of anti-PD-1 immunotherapies by reducing extracellular matrix stiffness and improving immune cell infiltration [39].

ITGA5 overexpression frequently correlates with poor prognosis in gastrointestinal tumors and facilitates tumor invasion via the FAK/AKT pathway. It is also implicated in immune cell infiltration as well as poor survival outcomes in various cancers, including gastric and colorectal tumors [40]. It has also been reported that ITGA5 expression is associated with lower overall survival and recurrence-free survival rates in laryngeal squamous cell carcinomas [41]. Furthermore, ITGA5 has been shown to be an important biomarker in predicting temozolomide and bevacizumab resistance in gliomas [42]. It has also been reported that high ITGA5 expression is associated with high immune cell infiltration in gliomas, and these patients may benefit from immune checkpoint blockade [43].

The HIF-1α/miR-142-3p/LOX/ITGA5 pathway has been studied in the context of chemotherapy resistance, particularly in triple-negative breast cancers. LOX inhibition has been reported to downregulate ITGA5 expression, increase extracellular collagen cross-linking and enhance drug penetration [21]. Our findings suggest that the increased HIF-1α, LOX and ITGA5 expression in the tumor microenvironment is associated with decreased PFS, potentially reflecting resistance to treatment. Patients with KRAS, NRAS or BRAF mutations and moderate-to-strong triple HIF-1α, LOX and ITGA5 expression exhibited worse pathological features, including a lower proportion of stage N0 or absence of lower rates of LVI. These findings highlight the prognostic role of mutations and hypoxia-associated protein interactions in tumor progression and metastasis.

Pathological biomarkers are essential for predicting treatment response and guiding therapy. In addition to CT or anti- EGFR therapies aimed at inhibiting tumor cell proliferation and survival, targets should be set to control the microenvironment which directs the cell’s mobility and provides a favorable environment for metastasis. Our study comprehensively examined the hypoxia pathway in the tumor microenvironment, focusing on the expression of HIF-1α, LOX and ITGA5 and their prognostic implications. The association of these proteins with decreased PFS suggests that therapies targeting this axis should be prioritized. Molecules targeting the HIF-1α, LOX and ITGA5 pathway are under development, with pre-clinical evidence highlighting the role of miRNAs in predicting CT resistance has been pre-clinically assessed. In addition, the correlated expression of these proteins in ECM indicates that the inhibition of any of these molecules may inactivate the entire pathway.

Limited studies have explored the relationships between tumor mutation status, hypoxia and tumor stroma in colon cancer [19,23,33,36]. Our findings indicate that stromal HIF-1α, LOX and ITGA5 expression is associated with PFS risk, regardless of mutation status. This highlights the potential significance of these proteins in tumor microenvironment-driven cancer progression. The activated KRAS oncoprotein triggers the MAPK, PI3K/AKT and JAK2/STAT3 HIF-1 signaling pathways, promoting HIF-1α and HIF-2α transcription and expression [44]. LOX expression is upregulated by PI3K/Akt activation and the concurrent accumulation of HIF-1α [23]. Collectively, RAS inhibitors exert antiproliferative and antimetastatic effects via the Ras-PI3K-Akt-HIF-1α-LOX signaling axis. In accordance with these findings targeting extracellular LOX or developing combination therapies with HIF inhibitors may offer a novel and efficient approach to improve CRC treatment outcomes [20].

Understanding the relationships between tumor stroma-associated proteins, hypoxia, and mutation status is expected to provide valuable insights for therapeutic strategies. Closely linked to HIF, targeting hypoxia presents a promising therapeutic strategy to halt the progression of various cancers and improve long-term survival outcomes for patients [45]. The antitumor activities of HIF-1 inhibitors have been well demonstrated in preclinical studies [46]. Despite the limited efficacy of the various HIF-1 inhibitors used in clinical trials, vorinostat has been reported to exert clinically recognizable benefits in the treatment of melanoma [47]. To improve the therapeutic efficacy of HIF inhibitors and reduce drug resistance and cancer-related pain, the development of combination therapies may be necessary. Further preclinical and clinical studies are warranted in order to determine the most promising HIF-1 inhibitors for combination therapies [17,48]. ITGA5 is overexpressed in pancreatic cancer-associated stroma and activated pancreatic stellate cells (PSCs) and mediates interactions between pancreatic cancer cells (PCCs) and PSCs. Targeting ITGA5 has shown promise in disrupting PCC-PSC interactions, thereby representing a potential therapeutic avenue for pancreatic cancer [49].

The LOX family has emerged as an appealing target for stromal modulation in solid tumors due to their roles in collagen crosslinking, stabilization and involvement in tumor desmoplasia. Chitty et al. have demonstrated that pan-lysyl oxidase inhibition significantly reduces chemotherapy-induced pancreatic tumor desmoplasia and stiffness, both in vitro and in vivo [50]. Cetin et al. have identified bi-thiazole derivatives as potent LOX inhibitors through a robust screening of drug-like molecules, incorporating cell- and recombinant protein-based assays. Their findings highlighted a highly potent bi-thiazole inhibitor which is capable of rewiring the collagen architecture and enhancing the chemotherapeutic response in triple-negative breast cancer [51].

This study has some limitations. First, the retrospective design may have introduced inherent biases, potentially affecting the validity of the findings. Second, the relatively small sample size (*n* = 100) limits the generalizability of the results to broader populations. Third, the study lacks external validation or comparison with independent cohorts, which constrains the robustness of the conclusions. Future research should focus on validating these findings using larger and more diverse datasets in order to enhance their applicability and reliability. 

## 6. Conclusions

This study highlights the prognostic significance of HIF-1α, LOX and ITGA5 expression in the tumor microenvironment, particularly in the context of KRAS/NRAS/BRAF mutation status. Although the number of cases in our study on the tumor mutation profile and tumor microenvironment in colorectal cancers was limited, significant results were still obtained in terms of addressing HIF-1α, LOX and ITGA5 expression in the tumor microenvironment and histopathological and clinical prognostic parameters in a broad framework and identifying significant relationship with progression-free survival risk, likely reflecting hypoxia-driven resistance mechanisms. More comprehensive studies examining the relationship between HIF-1α, LOX and ITGA5 proteins expressed in the tumor microenvironment/stroma or molecules that may have potential biomarker and targetable properties, as well as the molecular and genetic features of the tumor microenvironment, will contribute to the clarification of the biological behavior of the tumor, thus guiding the development of the treatments or alternatives. Additionally, further exploration of the HIF-1α, LOX and ITGA5 axis in various cancer types and its therapeutic targeting will provide deeper insights into its roles in cancer progression and resistance to treatment.

## Figures and Tables

**Table 1:** Mutation frequencies and MMR status of the study cases.

Mutation (KRAS/NRAS/BRAF)	Frequency *n* (%)
WILD	55 (% 55.0)
KRAS	30 (% 30.0)
NRAS	4 (% 4.0)
BRAF	11 (% 11.0)
Mutation	
WILD	55 (% 55.0)
KRAS A146X	5 (% 5.0)
KRAS A59T	1 (% 1.0)
KRAS G12X	17 (% 17.0)
KRAS G13D	7 (% 7.0)
NRAS G12X	1 (% 1.0)
NRAS Q61K	3 (% 3.0)
BRAF V600E/Ec	11 (% 11.0)
MMR (mismatch repair)	
proficient	87 (% 87.0)
deficient	13 (% 13.0)

**Table 2:** Progression-free survival times calculated using the Kaplan–Meier method and comparison of groups using the log-rank test.

Variables	N	Progression	Median Survival (Range)	*p* Value
Patients	40	28	11.07 (8.4–14.09)	
Gender				
Female	19	17	12.30 (7.76–16.84)	0.016
Male	21	11	25.95 (17.29–34.60)	
N stage				
N0	12	7	26.43 (16.58–6.28)	0.070
N1–N2	28	21	15.39 (9.62–21.15)	
KRAS mutation				
Negative	26	17	19.88 (12.70–27.05)	0.591
Positive	14	11	16.90 (10.33–23.46)	
BRAF mutation				
Negative	36	24	20.74 (14.91–26.58)	0.001
Positive	4	4	5.70 (3.74–7.67)	
HIF-1α tumor				
Negative	36	24	20.70 (14.84–26.56)	0.030
Positive	4	4	6.75 (0.72–12.79)	
HIF-1α microenvironment				
Weak	17	10	26.20 (17.59–34.79)	0.013
Moderate/strong	23	18	12.41 (8.05–16.76)	
ITGA5 microenvironment				
Weak	19	11	25.12 (16.76–33.54)	0.018
Moderate/strong	21	17	12.22 (7.75–16.69)	
LOX microenvironment				
Weak	20	13	23.57 (16.24–30.90)	0.05
Moderate/strong	20	15	11.99 (6.75–17.24)	
LOX tumor				
Low	22	16	20.51 (13.31–27.71)	0.572
High	18	12	14.71 (9.14–20.28)	
All IHC positive				
No	25	16	23.29 (16.44–30.14)	0.014
Yes	15	12	9.93 (4.95–14.9)	
Metastatic first-line treatment				
CT + Anti-VEGF	21	15	16.05 (10.42–21.68)	0.023
CT + Anti-EGFR	14	8	26.18 (16.53–35.83)	
CT	5	5	7.80 (5.90–9.70)	

N: Lymph node status; HIF-1α: hypoxia-inducible factor 1 alpha; ITGA5: integrin alpha 5; LOX: lysyl oxidase; IHC: immunohistochemistry; CT: chemotherapy for the log-rank test; a *p*-value less than 0.05 was considered statistically significant.

**Table 3:** Cox regression model results for progress-free survival in colorectal cancer patients.

Clinicopathologic Feature	HR (95% CI)	*p*-Value
Gender		
Female vs. male	0.35 (0.14–0.84)	0.020
Mutation Positive		
Any of KRAS, NRAS, or BRAF	0.86 (0.22–3.35)	0.838
BRAF Mutation		
Negative vs. positive	1.74 (0.36–8.32)	0.487
All IHC-Positive		
Triple positive (HIF-1α/LOX/ITGA5) with moderate to strong intensity vs. all others	4.32 (1.29–14.48)	0.018
Treatment		
CT + anti-VEGF		0.021
CT + anti-EGFR	0.32 (0.66–1.64)	0.175
CT	3.40 (1.01–11.48)	0.048

HR: hazard ratio, CI: confidence interval. A *p*-value less than 0.05 was considered statistically significant.

**Table 4:** Patient and tumor characteristics according to mutation and IHC status.

Variables	Mutation and IHC Positivity		*p* Value
	No (*n* = 85)	Yes (*n* = 15)	
Gender			
Female	30 (% 35.3)	5 (% 33.3)	0.883
Male	55 (% 64.7)	10 (% 66.7)	
Tumor size	4.5 (0.2–15)	5 (1.9–9)	0.361 *
Metastatic lymph node number (median)	1	3	0.033 *
Differentiation			
Well	20 (% 23.5)	0 (% 0.0)	0.026
Moderate–poor	65(% 76.5)	15 (% 100)	
T stage			
T2–3	60 (% 70.6)	7 (% 46.7)	0.069
T4	25 (% 29.4)	8(% 53.3)	
N stage			
N0	36 (% 42.4)	2 (% 13.3)	0.028
N1–2	49 (% 57.6)	13 (% 86.7)	
Stage			
Stage I–III	46 (% 54.1)	5 (% 33.5)	0.138
Stage IV	39 (% 45.9)	10 (% 66.7)	
LVI			
Negative	30 (% 35.3)	1 (% 6.7)	0.021
Positive	55 (% 64.7)	14(% 93.3)	
PNI			
Negative	54 (% 63.5)	3 (% 33.3)	0.151
Positive	31 (% 36.5)	6 (% 66.7)	
MMR			
Proficient	75 (% 88.2)	12 (% 80)	0.303
Deficient	10 (% 11.8)	3 (% 20)	

N: lymph node status, T: tumor, LVI: lymphovascular invasion, PNI: perineural invasion lysyl oxidase, IHC: immunohistochemistry, CT: chemotherapy, MMR: mismatch repair. A *p*-value less than 0.05 was considered statistically significant. Mann–Whitney U test for non-parametric comparisons; all other tests are chi-square tests. * Mann–Whitney U test.

## Data Availability

The datasets used and/or analyzed during the current study are available from the corresponding author upon reasonable request.
